# Impact of Andropause on Multiple Sclerosis

**DOI:** 10.3389/fneur.2021.766308

**Published:** 2021-11-05

**Authors:** Maria C. Ysrraelit, Jorge Correale

**Affiliations:** Fundación para la Lucha contra las Enfermedades Neurológicas de la Infancia (FLENI), Buenos Aires, Argentina

**Keywords:** andropause, testosterone, aging, sex hormones, multiple sclerosis

## Abstract

Andropause results from the natural decrease in testosterone levels that occurs with age. In contrast to menopause, which is a universal, well-characterized process associated with absolute gonadal failure, andropause ensues after gradual decline of both hypothalamic-pituitary-gonadal axis activity, as well as of testicular function, a process which usually develops over a period of many years. Increasing evidence on greater risk of Multiple sclerosis (MS) associated with lower testosterone levels is being reported. Likewise, epidemiological studies have shown a later age of onset of MS in men, relative to women, which could perhaps respond to the decline in protective testosterone levels. In this review, we will discuss the role of androgens in the development and function of the innate and adaptive immune response, as well as in neuroprotective mechanisms relevant to MS. Testosterone effects observed in different animal models and in epidemiological studies in humans will be discussed, as well as their correlation with physical disability and cognitive function levels. Finally, published and ongoing clinical trials exploring the role of androgens, particularly at key stages of sexual maturation, will be reviewed.

## Introduction

Multiple sclerosis (MS) is a demyelinating immune-mediated disease of the central nervous system (CNS) universally more prevalent in women than in men, a phenomenon shared with several other autoimmune diseases. Indeed, different studies have reported greater MS prevalence in females, with the current female/male ratio estimated to be 3:1 (1). Important gender differences in inflammatory activity and progression of disease have also been observed. Male patients not only develop disease later, they experience less relapses. However, they also accumulate disability faster, reach milestones more rapidly, and show poorer recovery after initial exacerbations, compared to females (2). These findings have led to extensive studies on differences between the immune and nervous systems of men and women, in response not only to their specific gonadal hormones, but also to genetic factors, exposure to environmental factors and varying lifestyles (3). It has been hypothesized that the natural age-related decline in testosterone, the main male sex hormone, may play a role in these gender-related differences in MS prevalence and in clinical characteristics.

Several studies have highlighted the modulatory role of female sex hormones on MS disease activity during different stages of reproduction such as puberty, pregnancy and menopause. However, less is known about the impact of reproductive senescence in men. In contrast to menopause, which is a universal, well-characterized process associated with absolute gonadal failure, andropause is characterized by a gradual decline in hypothalamic-pituitary-gonadal axis activity, as well as decreased testicular function, occurring over a period of many years. Concentrations of bioavailable testosterone can decrease by as much as 50% between the ages of 25 and 75 years (4). Given that in men there is no abrupt hormonal cutoff, or clear period of symptomatic change, some authors argue that the term “andropause” or “male menopause” is inappropriate, and that the phenomenon should be called late-onset hypogonadism (LOH) or age-related androgen decline (5). Dehydroepiandrosterone (DHEA), an adrenal precursor to more potent androgens and estrogens, as well as its metabolite DHEA sulfate (DHEA-S) also decline with age, at a rate of 3% per year, 3 times faster than testosterone, falling to one-third of serum concentrations by age 70 (6).

In this review we will discuss the role of androgens in the development and function of the innate and adaptive immune response, as well as neuroprotective mechanisms relevant to MS. Testosterone effects observed in different animal models and epidemiological studies in humans will be analyzed, as well as their correlation with physical disability and cognitive function levels. Finally, we will review published and ongoing clinical trials exploring the role of androgens, particularly at different stages of sexual maturation.

## Effects of Androgens on Immune Function

Andropause appears to contribute, at least in part, to immunosenescence, and consequently to progression of disability in MS. Immunosenescence is an age-associated decline in function of both the adaptive and innate immune systems. MS patients may experience premature onset of this phenomenon (7). In parallel, specific effects of androgens include a shift from Th1 to Th2 phenotype, based on increased production of IL-5 and IL-10, and decreased pro-inflammatory cytokines including IFN-γ, TNFα, IL-1, IL-6, and IL-17. Testosterone also reduces lymphocyte proliferation and differentiation and may suppress immunoglobulin production (8). Supra-physiological doses of testosterone also inhibit cytotoxic NK cell activity (9, 10). Therefore, androgens should, in principle, be considered anti-inflammatory hormones. In the CNS, dihydrotestosterone inhibits LPS-induced release of proinflammatory factors, including TNF-α, IL-1β, IL-6; iNOS, COX-2, NO, and PGE2 in BV2 cells and primary microglia cells, through suppression of TLR4-mediated NF-κB and MAPK p38 signaling pathways, thus protecting neurons from inflammatory damage induced by activated microglia (11). Similar changes have been observed in experimental *in vivo* models. In fact, castration of male animals has had detrimental effect on susceptibility to, and severity of EAE (12). MBP-specific T lymphocytes derived from the spleen of male animals during the effector phase of adoptive EAE, produced significantly higher levels of IL-10 (13), and treatment with testosterone was protective, an effect linked to androgen-mediated Th2 bias, as suggested by the IFNγ/IL-10 ratio (12, 13).

Similar to testosterone, DHEA inhibits transcription factor NF-κB, and suppresses secretion of IL-1β, TNF-α, and IFN-γ (14). In animal models, DHEA decreases T cell response and shows anti-inflammatory effect on microglia and astrocytes, ameliorating EAE severity and inflammation (15, 16).

In the thymus, the autoimmune regulator (Aire) gene prevents autoimmunity by promoting self-antigen expression in medullary thymic epithelial cells, such that developing T cells that recognize these self-antigens within the thymus, undergo clonal deletion. Androgens recruit androgen receptors to Aire promoter regions, enhancing Aire transcription. Thus, androgen levels in males may increase Aire expression to a degree that protects against autoimmunity. In line with this, in EAE mice, androgen administration as well as male gender, protected against autoimmunity in an Aire-dependent manner, indicating that control of intrathymic Aire-mediated tolerance mechanisms by androgens, contributes to explain gender-related differences observed in MS (17).

## Neuroprotective Mechanisms

Testosterone crosses the blood-brain-barrier, directly influencing neuronal cells (18, 19). The main effects of testosterone on the CNS are summarized in Table 1. Similarly, in humans, DHEA has demonstrated neuroprotective effects, increasing neurite growth, promoting neurogenesis and neuronal survival, influencing apoptosis, and catecholamine synthesis and secretion, as well as exerting antioxidant effects (6).

**Table 1:** Main neuroprotective effects of androgens.

**Effects of testosterone on the CNS**	**References**
Improves survival of human neurons and astrocytes, inhibiting generation of reactive oxygen and nitrogen species.	(20, 21)
Upregulates of neuroglobin secretion by astrocytes and microglia after injury, glucose deprivation, and kainic acid toxicity.	(22–25)
Increases expression of neurotrophic factors such as brain derived neurotrophic factor (BDNF) which activates brain neurogenesis, dendritic spine maturation and modulates motor neuron morphology.	(26–28)
Stimulates of neural plasticity and neural differentiation	(29)
Promotes synaptic density and increase the growth of neurites	(30)
Increases connectivity of hypothalamic neurons	(31)
Reduces reactivity of astrocytes and reactive microglia following brain injury	(32)
Delays the aging process	(33)
Preserves excitatory synaptic transmission in the hippocampus during EAE	(34)

Recent studies have shown that testosterone may also play an important role in myelination processes. Indeed, after lysolecithin-induced demyelination, testosterone favors astrocyte recruitment and spontaneous oligodendrocyte-mediated remyelination (35). Similarly, castration of male animals results in decrease in myelination in the corpus callosum, both under normal conditions and after long term administration of cuprizone. These processes are reversed following exogenous testosterone administration (36).

In line with these findings, other studies have shown that testosterone, *via* specific involvement of androgen receptors, induces proliferation and differentiation of oligodendrocyte precursors (OPCs), as well as activation and proliferation of astrocytes and microglial cells (36).

## Clinical Findings

Andropause or LOH refers to the gradual and lifelong decline in serum testosterone concentration and testicular function that occurs with aging. Healthy older men will experience approximately 40% reduction in total Leydig cell mass over time. LOH will also likely be influenced by comorbidities associated with aging and the development of chronic illnesses, including obesity, diabetes, cardiovascular disease, and inflammatory disorders, all associated with accelerated aging-related testosterone decline. Presentation of MS symptoms in older patients will be further impacted by these events (37, 38).

Clinical characteristics of andropause include diminished sexual desire and erectile capacity, decreased intellectual activity, fatigue, depression, loss of muscle mass and body hair, anemia, decrease in bone mineral density resulting in osteoporosis, and increased visceral fat and obesity (39). These symptoms may overlap with the effects of aging, worsening the motor disability, fatigue, cognitive decline and psychiatric symptoms caused by MS (19).

**Figure 1:**
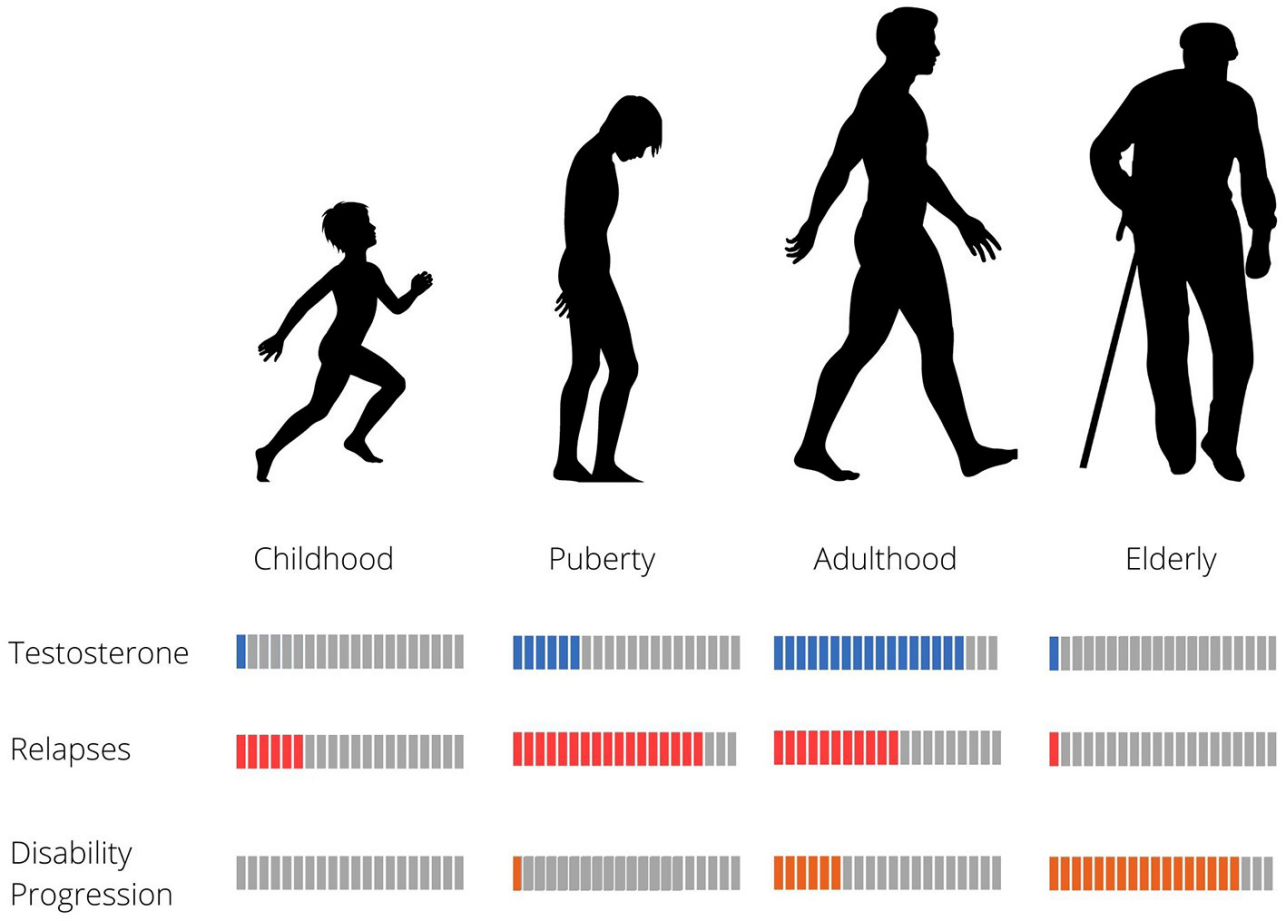
Relapse rate, disability progression and testosterone levels in relation to age in MS males.

Both MS symptoms as well as their severity also appear to differ between males and females. Men present later onset of disease (40, 41) and experience less frequent relapses with poorer recovery (42, 43). They show faster progression (Malik Neurology, 2014), worse outcomes (42) more cerebellar involvement, as well as greater cognitive impairment (42, 44). In line with these clinical findings, MRIs from men with MS show less inflammation (40), more gray matter atrophy (44) and more T1 lesions (45). Intriguingly, these clinical findings are not observed in pediatric MS cases prior to puberty (46), or in women with MS onset during menopause (around 50 years of age on average in Western societies), suggesting a more complex interplay between hormonal mechanisms related to reproductive senescence or aging, and the course of the disease (47). Young MS patients show slower progression of disability than adults or late onset MS patients, but experience relapses more frequently (48, 49). Risk of relapse seems to decrease continuously with time until patients reach the age of 35. In contrast, disability worsening remains stable from childhood to about 32 years of age, and then increases sharply after the age of 45 (50). Typical age of MS onset in men is around 40, coinciding with the physiological age-related decline in androgen levels, suggesting loss of testosterone could contribute to development of MS. Overlap with other phenomena related to aging, such as immunosenescence could also influence patient symptom profiles. Effects of androgen levels and aging in men with MS are summarized in Figure 1.

Several studies have found lower testosterone levels in men with MS, compared to healthy age-matched subjects (51–53). In a cohort of 96 men with relapsing remitting MS, mean age of 40 years and disease duration <10 years, 39% of patients were hypogonadal with no compensatory rise in luteinizing hormone, suggesting central hypogonadism (54). Interestingly, the authors reported a correlation between low testosterone levels and disability (54). Other studies however, were not able replicate these findings (53).

An analysis of linked national Hospital Episode Statistics from England reported a strong positive association (5-fold elevation) between testicular hypofunction, as a proxy for low testosterone levels, and subsequent risk of MS in males (55).

Recently, increased risk of MS was reported in transgender individuals receiving estrogens and anti-androgens (56), suggesting influence of feminizing hormones, or low testosterone levels on risk of disease, providing further evidence of the importance of sex hormones in MS pathophysiology.

## Potential Use of Testosterone for Ms Treatment

Testosterone replacement therapy is commonly indicated in aging and hypogonadal men. Hormonal supplementation induces virilization, improved libido and energy, increased muscle strength and fat-free mass, and strengthens bone density. Its use requires monitoring of prostate-specific antigen, as well as of hematocrit levels.

In a pilot clinical trial with cross-over design, 10 RRMS men aged <65 years, were treated with 100 mg of testosterone (6 months observation followed by 12 months of treatment). Treatment resulted in improvement of cognitive performance, and slowing of brain atrophy, with no significant effect on gadolinium-enhancing lesions (Gd) (57). Subsequent evaluations of this same study have shown testosterone treatment decreased CD4+T cells and IL-2 production, and increased NK cells as well as TGF-β1 secretion (8). Furthermore, voxel-based morphometry of the brain showed not only less global atrophy, but also significant increase in gray matter in a cluster in the right frontal cortex (58).

TOTEM RRMS (NCT 03910738) is a phase 2, multicenter, randomized placebo controlled, double-blind trial carried out in 40 testosterone-deficient men with relapsing-remitting MS, which aims to prevent MS progression. Patients will be randomized into two groups, to receive either testosterone undecanoate or placebo over a period of 66 weeks. All patients will be treated with natalizumab during the trial. The primary outcome is to measure the neuroprotective and remyelinating effects of testosterone using tensor diffusion imaging techniques and thalamic atrophy analyzes. Secondary outcomes include use of conventional MRI sequences as well as clinical parameters to assess cognition, fatigue, quality of life, impact on work activity and anxiety/depression. Recruitment is expected to end around September 2021 (59).

The main clinical trials using testosterone are summarized in Table 2. These trials underline the potential use of testosterone as an immunomodulatory, neuroprotective and remyelinating molecule. Importantly, testosterone did not cause significant side effects in any of these trials, suggesting this treatment could represent a safe adjunctive therapy for MS and other neurodegenerative diseases (19).

**Table 2:** Main clinical trials using testosterone in MS.

**Study Title**	**Interventions**	**Design**	**Main Outcome**	**Status**	**Comments**
Testosterone Treatment for Multiple Sclerosis (NCT00405353)	Androgel: 10 grams of gel containing 100 mg of testosterone	Pilot cross-over10 patients	Brain atrophy rate and Cognitive testing	Completed	One year of follow-up, shows improvement in cognitive performance and slowing brain atrophy.
Oral Testosterone for Fatigue in Male Multiple Sclerosis Patients (NCT01516554)	Testosterone undecanoate vs. placebo	Randomized controlled cross-over	Change in Modified Fatigue Impact Scale	Terminated due to poor recruitment	—–
Testosterone Treatment for Erectile Dysfunction and Multiple Sclerosis (NCT04601233)	Testosterone 75 mg sub cutaneous using auto injector	Single open labelEstimated enrollment: 20 patientsIntervention period: 12 weeks	Self-reported erectile function measured by: ADAM Score SHIM Score MSHQ-SF.	Not yet recruiting	—-
TOTEM RRMS: TestOsterone TreatmEnt on Neuroprotection and Myelin Repair in Relapsing Remitting Multiple Sclerosis (NCT03910738)	Testosterone Undecanoate 1,000 mg vs. Placebo	Multicentric, randomized, parallel groups DBPC, Phase 2Estimated enrollment: 40 patientsIntervention period 66 weeks	Change on MRI binary criterion combining thalamic atrophy and modification in transverse diffusivity.	Recruiting	—–

*ADAM Score, Androgen Deficiency in the Aging Male; DBPC, Double blind placebo controlled; SHIM Score, Sexual Health in Men; MSHQ-SF, Sexual Health Questionnaire short form*.

## Conclusions and Future Perspectives

Age is one of the major determinants of the clinical course of MS. Aging mechanisms will likely affect multiple clinical aspects of the disease, as well as influence underlying pathological mechanisms, immunological changes, and treatment efficacy.

Notably, transition from RRMS to more progressive disease phases will overlap with the naturally occurring age-related decline of androgens in men, and with menopause in women. Clinical findings already suggest specific association between reproductive senescence and MS progression. We have described the influence of testosterone on MS and its potential effects on immunosenescence as well as on neuroprotection. However, further longitudinal studies on disease activity in diverse populations of men with MS are needed. Available information regarding DMTs in older men suffering from andropause and MS is limited, as clinical trials often restrict enrollment to those aged < 55 years. Understanding the complex interplay between reproductive senescence and the course of MS is of critical importance to elucidate mechanisms driving disease progression in older patients, and minimize progression of disability.

## Author Contributions

All authors listed have made a substantial, direct and intellectual contribution to the work, and approved it for publication.

## Conflict of Interest

The authors declare that the research was conducted in the absence of any commercial or financial relationships that could be construed as a potential conflict of interest.

## Publisher's Note

All claims expressed in this article are solely those of the authors and do not necessarily represent those of their affiliated organizations, or those of the publisher, the editors and the reviewers. Any product that may be evaluated in this article, or claim that may be made by its manufacturer, is not guaranteed or endorsed by the publisher.
